# Aberration-Based Quality Metrics in Holographic Lenses

**DOI:** 10.3390/polym12040993

**Published:** 2020-04-24

**Authors:** Tomás Lloret, Víctor Navarro-Fuster, Manuel G. Ramírez, Marta Morales-Vidal, Augusto Beléndez, Inmaculada Pascual

**Affiliations:** 1 Departamento de Óptica, Farmacología y Anatomía, Universidad de Alicante, Carretera San Vicente del Raspeig s/n, 03690 San Vicente del Raspeig, Spain; tll3@alu.ua.es (T.L.); ramirez@ua.es (M.G.R.); a.belendez@ua.es (A.B.); 2 Departamento de Física, Ingeniería de Sistemas y Teoría de la Señal, Universidad de Alicante, Carretera San Vicente del Raspeig s/n, 03690 San Vicente del Raspeig, Spain; victor.navarro@ua.es; 3 Instituto Universitario de Física Aplicada a las Ciencias y las Tecnologías, Universidad de Alicante, Carretera San Vicente del Raspeig s/n, 03690 San Vicente del Raspeig, Spain; marta.morales@ua.es

**Keywords:** holographic lenses, low toxicity photopolymer, optical quality, aberrations

## Abstract

Aberrations and the image quality of holographic lenses were evaluated by a Hartmann–Shack (HS) wavefront sensor. Two lenses, one recorded with a symmetrical configuration and the other with an asymmetrical one, were stored in a photopolymer called Biophotopol. Each was reconstructed with two different wavelengths, 473 nm and 633 nm. Different metrics were applied to determine and quantify the aberration of the lenses (Zernike coefficients, Seidel coefficients, Marechal tolerances, root-mean-square (RMS), peak to valley, critical fraction of the pupil), and the quality of the image they provided (Strehl ratio, entropy, cutoff frequency, modulation transfer function (MTF), and area under the MTF). Good agreement between the metrics related to optical quality was obtained. The negative asymmetric holographic lenses had less aberration than the positive symmetric ones.

## 1. Introduction

Holography is a method of producing three-dimensional images in two stages: recording and reconstruction. Recording is based on photoinduced changes in holographic material, generating a refractive index modulation that corresponds to the spatial distribution of the light intensity generated by the interference of the recording beams. Reconstruction is based on the diffraction phenomenon [1]. Holography is a very interesting technique because it allows the information to be encoded in a recording material and has evolved considerably, thanks to the improvement of holographic materials such as photopolymers [2]. One of the most relevant holographic applications is holographic optical elements (HOEs) [3,4]. A HOE can transform an incident optical beam as a conventional lens with the advantages of obtaining high optical power in a thin substrate [5]. Moreover, the ease with which the element can be coupled for any type of manipulation and by multiplexing two or more HOEs [6,7] can put together various functions in a single substrate according to its narrow-band frequency and high diffraction efficiency characteristics. 

HOEs are essential optical elements used in many systems, credit cards, filters, screens, projection systems, couplers, and storage [8,9,10,11,12]. HOEs have evolved together with the development of different fields such as photonics, communications, and information processing. Currently, holographic lenses (HLs) are part of optical imaging systems with applications primarily in augmented reality (AR) [13,14,15,16,17]. A single HL working in an optical system presents aberrations, so it is necessary to know and quantify these aberrations [18]. When HLs are included in AR optical systems such as head mounted displays (HMD), the aberration correction must be carried out [15,16,17]. Traditionally in holography, aberrations have been evaluated theoretically [19,20,21,22] and experimentally by obtaining the point spread function (PSF) of the image by a charged coupled device (CCD) sensor [23,24]. However, in telescopes [25] or visual systems [26,27] in optometry and ophthalmology, aberrations have been studied in-depth by using aberrometers. One of the simplest and well-known aberrometer is the Hartmann–Shack (HS) wavefront sensor [28]. The HS sensor is based on a Hartmann screen that has holes distributed uniformly to obtain an impact diagram in the image plane. To avoid low illumination conditions, a lens before each hole was added by Shack, thus improving the measurements [28]. In this paper, the HS wavefront sensor was used to quantify aberrations in holographic lenses for the first time. Furthermore, the HS sensor has been used previously to study coherent holographic imaging [29] in a lensless system. 

The material in which the HLs are to be recorded is an important decision since the aberrations must be influenced by it. There are many different materials for recording HLs [2] such as dichromated gelatin [30,31], silver halide emulsions [32,33], photopolymers [34], photorefractive materials [11], and photoresist [35]. Out of all of these materials, photopolymers are the most versatile material. Photopolymers can be modified in terms of both design and composition. In 1969, Close et al. [36] were the first to use them to make HOEs. Low cost, variable thickness, flexibility, self-processing capabilities [37], high energetic sensitivity, good dimensional stability, sharp angular selectivity and large dynamic range are some of the other interesting properties. The importance of photopolymers is growing extraordinarily [38], and a great variety of photopolymer materials has been used enormously in optical applications [39]. 

Usually, holographic photopolymers contain acrylamide [40,41,42,43], which is a toxic compound. The latest trends in holographic materials include photopolymers with low toxicity [44,45,46], environmental compatibility [47,48], and good recycling properties. Our research team has developed a low toxicity photopolymer called “Biophotopol” for recording HOEs, in general such as HLs, for an extensive range of optical applications [7,49,50,51].

The aim of this work was to study the optical and image quality of different types of HLs fabricated in Biophotopol using a HS wavefront sensor to obtain the aberration parameters needed to quantify the HL quality by different metrics [52,53,54]. Metric analysis can be undertaken related to aberrations or image quality. Zernike coefficients, Seidel coefficients, Marechal tolerances, root-mean-square (RMS), peak to valley, and critical fraction of the pupil are metrics related to aberrations. Metrics related to the quality of the image are the Strehl ratio, entropy, cutoff frequency, modulation transfer function (MTF), and area under the MTF. All are described in detail in the next section.

## 2. Materials and Methods 

### 2.1. Material Composition

The component concentrations of the low toxicity photopolymer Biophotopol have been extensively studied in previous papers [7,50]. A solution composed of sodium acrylate (NaAO) as the polymerizable monomer (NaAO was generated in situ through a reaction of acrylic acid (HAO) with sodium hydroxide (NaOH) in a 1:1 proportion), sodium salt 5′-riboflavin monophosphate (RF) as a sensitizer dye, triethanolamine (TEA) as the co-initiator, and polyvinyl alcohol (PVA) as an inert binder polymer (*M*_w_ = 130,000 g/mol, hydrolysis grade = 87.7%). All compounds were purchased from Sigma-Aldrich Quimica SL (Madrid, Spain) and used in a water prepolymer solution. The optimized concentrations were 0.39 M, 1.0·10^−3^ M, 9.0·10^−3^ M, and 13.5 wt/v % for NaAO, RF, TEA, and PVA, respectively, (or 13.5, 3.71, 0.13 and 0.06 wt/v % for PVA, NaAO, TEA, and RF, respectively). The prepolymer solution was deposited in square glass molds by the force of gravity (6.5 × 6.5 cm^2^) and left (about 24 h) inside an incubator (Climacell 111, Labexchange, Burladingen, Germany) at a controlled humidity and temperature (RH = 60% ± 5% and T = 20 ± 1 °C). The exposure must be done immediately [24]. After exposure, the holographic lenses were cured with a light-emitting diode lamp (LED) (13.5 W, 875 lm at 6500 K, Lexman) for 20 min in order to eliminate the residual dye. The final solid film had a physical thickness of around 200 µm.

### 2.2. Holographic Process

**Figure 1:**
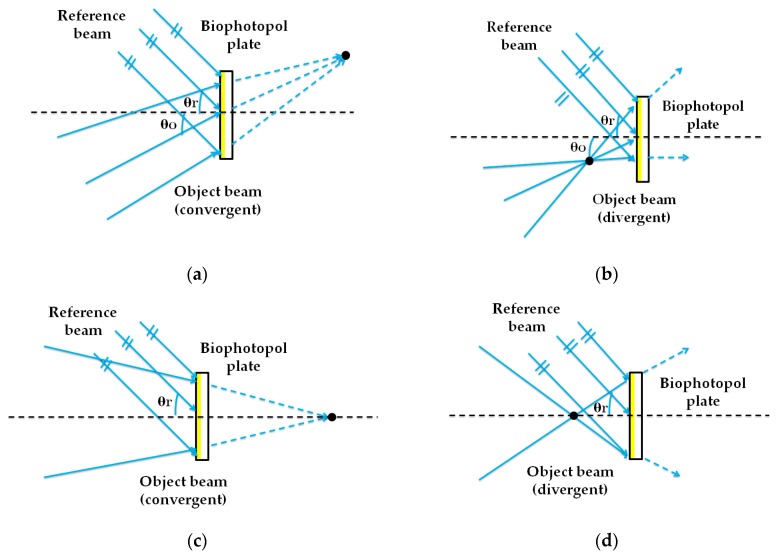
Holographic storage recording process (488 nm) with symmetric (**a**) and (**b**), and asymmetric (**c**) and (**d**) recording beams; and with positive (**a**) and (**c**), and negative (**b**) and (**d**) focal points.

**Figure 2:**
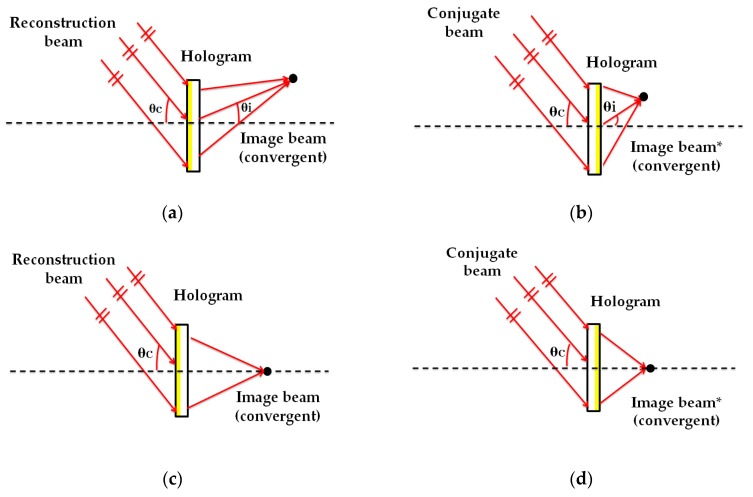
Holographic storage reconstruction (473 nm and 633 nm) of symmetric (**a**) and (**b**), and asymmetric (**c**) and (**d**) geometry; and with positive (**a**) and (**c**), and negative (**b**) and (**d**) focal points.

**Figure 3:**
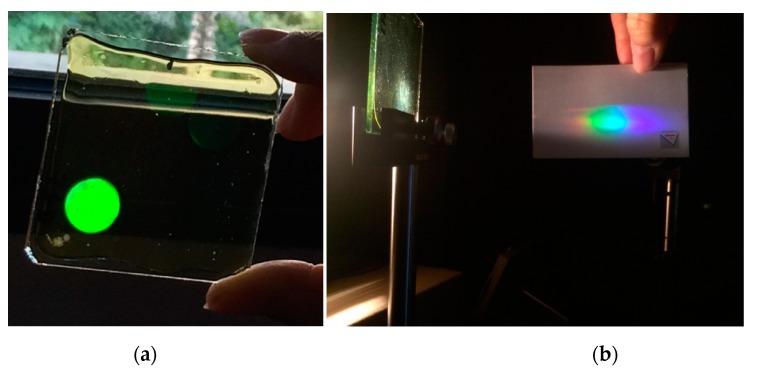
(**a**) Photography of a negative asymmetric volume phase transmission holographic lens observed by reflection illuminated with daylight. A single-color image is observed because it shows by reflection the area in which the evaluated holographic lens is located, (**b**) chromatic dispersion is watching in the same holographic lens working by transmission with a white conjugate collimated beam.

HLs are object point holograms located at a certain distance from the recording material in a typical holographic setup [24]. In the recording stage, as seen in Figure 1, the HLs were obtained from the interference of a reference beam (plane beam) and an object beam, converging or diverging, depending on the HL type. The HLs evaluated were manufactured with symmetric (Figure 1a,b) and asymmetric (Figure 1c,d) recording beams and with positive (Figure 1a,c) and negative (Figure 1b,d) focal points. In the reconstruction stage (Figure 2), the positive HLs were reconstructed with a plane beam hitting the material face where the hologram is located (Figure 2a,c). Instead, the negative HLs were reconstructed with the same beam, but hit the holographic recording material on the opposite side where the hologram is located (Figure 2b,d). This is called a conjugated image when it refers to the image point reconstructed by this configuration, or a conjugated beam when it refers to the reconstructed beam entry onto the hologram on the opposite side. [4]. The angle of the reconstruction beam for the different lasers (473 nm and 633 nm) was calculated using Bragg’s law [55]. Table 1 and Table 2 show the object, reference, reconstruction, and image angles (o, r, c, i, respectively) and the focal length of the holographic lens ($f\prime_{HL}$) in each case. The diameter of the holographic lenses was 12 mm, but in this work, we only worked with the central 6 mm due to the limitation imposed by the complementary metal-oxide-semiconductor (CMOS) of the HS wavefront sensor. Considering the data in Table 1, the spatial frequency values varied from 1142 lines/mm to 1266 lines/mm. The central value was 1205 lines/mm. These values were the same for symmetric and asymmetric HLs. Figure 3 shows two pictures of a negative asymmetric volume phase transmission holographic lens evaluated in this paper. In Figure 3a, a lens was observed by reflection illuminated with daylight. A single-color image was observed because it shows by reflection the area in which the holographic lens is located. Figure 3b shows how the same holographic lens works by transmission with white light. The phenomenon of chromatic dispersion is watching when the HL is illuminated with a white conjugate collimated beam. The screen shows the image focal points for each color, where the red is closer to the lens (short focal distance) with a bigger reconstruction angle (see Table 2) and the blue is farther to the lens (large focal distance) with a lesser reconstruction angle (see Table 2).

### 2.3. Evaluation of the Aberrated Wavefront of HLs

**Figure 4:**
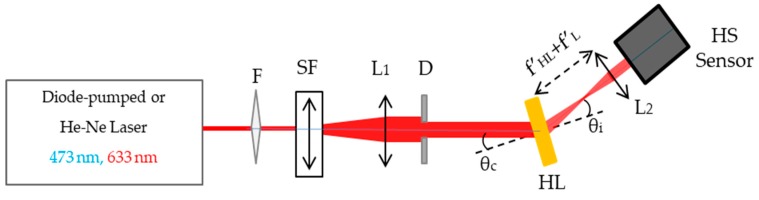
Experimental setup for the evaluation of the aberrated wavefront of holographic lenses. F: filter, SF: spatial filter, L: lens, D: diaphragm, HL: holographic lens, HS Sensor: Hartmann–Shack wavefront sensor.

The experimental holographic setup used to obtain the wavefront can be seen in Figure 4. To illuminate the HLs, a 473 nm diode-pumped laser—whose wavelength was close to the recording one (488 nm)—was used together with a 633 nm He–Ne laser. The beam, after being spatially filtered, was placed at the reconstruction angle ($\theta_{C}$) that matched the recording angle. The conjugate beam was used when the lenses evaluated were negative. The HL and L2, separated by a distance $f\prime_{HL}+f\prime_{L}$ formed an afocal lens system [55,56], from which the collimated emerging beam ends up hitting the HS wavefront sensor. It is important to note that the collimated beam that reached the HS sensor had previously been calibrated. In this case, the ideal wavefront was plane, thus the real wavefront coincided with the wavefront aberration. Table 2 shows the reconstruction geometry, image distance ($f\prime_{HL}$), and the reconstruction ($\theta_{c}$) and image ($\theta_{i}$) angles.

The wavefront sensor used in this work was a Hartmann–Shack WFS30-5C model from Thorlabs. This instrument is composed of a 1936 × 1216 pixel CMOS camera with an active area of 11.34 × 7.13 mm^2^, an array of microlenses with a pitch of 150 µm, and an effective focal length of 4.1 mm. These sensors are also characterized by having a distance between centers (from pixel to pixel) of 5.83 μm. Consequently, among the photoelements of a certain ∆x direction, the Nyquist frequency is defined as [57]:

(1) $$\xi_{Ny}=\frac{1}{2\Delta x}$$

where the value for this sensor is 85.76 lp/mm or using the conversion equations of [58], 351.6 cyc/deg. This value indicates that the HS sensor is not capable of detecting spatial frequencies higher than 351.6 cyc/deg.

### 2.4. Optical Quality Metrics Based on Aberrations

Optical quality metrics are metrics based on the aberrated wavefront emerging from the optical system. In this case, the metrics refer to the optical quality of the HLs studied. To this end, the aberrations in the exit pupil of the HLs were studied.

#### 2.4.1. Wave Aberration Function 

Historically it has been seen that the wave aberration function *W* can be mathematically represented as a sum of Taylor monomials (given in Cartesian coordinates). The appropriate combination of monomials allows the different optical aberrations to be obtained. Another option, in the case of optical systems with circular pupils, is to describe the aberrations in polar coordinates instead of Cartesian coordinates, since the former are easier for these systems.

The usual way to represent a point in the ${\mathbb{R}}^{2}$ plane is by rectangular coordinates (x,y), however, polar coordinates (r,θ) can be very useful in this plane. The transformation equations are:

(2) x=rcos(θ)y=rsin(θ)

where $r=\sqrt{x^{2}+y^{2}}$, r≥0, and 0≤θ≤2π. For optical systems with circular pupils of radius $r_{0}$, it is convenient to normalize the radial coordinate in the exit pupil of the system with respect to the $r_{0}$ radius. So, the new radial coordinate will be ρ=rr0.

Zernike polynomials are defined mathematically as a sequence of polynomials that are orthogonal with respect to the scalar product of functions on the unit disk [52,59,60,61].

The general form is

(3) $$Z_{n}^{m}\left(\rho ,\theta \right)=\{\begin{array}{c} N_{n}^{m}R_{n}^{\left|m\right|}\left(\rho \right)cos\left(m\theta \right)\;for\;m\geq 0\\ -N_{n}^{m}R_{n}^{\left|m\right|}\left(\rho \right)sin\left(m\theta \right)\;for\;m<0 \end{array}$$

where the subscript *n* denotes the degree of the radial polynomial and the superscript *m* denotes the angular frequency.

In addition, the explicit form of the radial polynomial, $R_{n}^{\left|m\right|}$, is defined as

(4) $$R_{n}^{\left|m\right|}\left(\rho \right)=\overset{\left(n-\left|m\right|\right)/2}{\underset{s=0}{\sum }}\frac{{\left(-1\right)}^{s}\left(n-s\right)!}{s!\left[\frac{\left(n+\left|m\right|\right)}{2}-s\right]!\left[\frac{\left(n-\left|m\right|\right)}{2}-s\right]!}\rho^{n-2s}$$

which is a polynomial of degree *n* containing the terms $\rho_{n},\;\rho_{n-2},\ldots ,\rho_{m}$.

On the other hand, the normalization factor, $N_{n}^{m}$, is defined as

(5) $$N_{n}^{m}=\sqrt{\frac{2\left(n+1\right)}{1+\delta_{m0}}}$$

where $\delta_{m0}$ is the Kronecker Delta.

The advantage of using Zernike polynomials is that any of the aberrations can be described as $C_{n}^{m}\cdot Z_{n}^{m}\left(\rho ,\theta \right)$, where $C_{n}^{m}$ are the Zernike coefficients, defined as expansion coefficients that depend on the location of the object point. The wave aberration function can be defined as a combination of several aberration terms and mathematically defined from Zernike polynomials as:

(6) $$W\left(\rho ,\theta \right)=\overset{k}{\underset{n=0,\;m=-n}{\sum }}\overset{n}{\underset{n-\left|m\right|=par}{\sum }}C_{n}^{m}\cdot Z_{n}^{m}\left(\rho ,\theta \right)$$

Only the contributions of spherical aberration, coma, and astigmatism were taken into account in this work and their coefficients are $C_{4}^{0}$, $\left(C_{3}^{1},C_{3}^{-1}\right)$, and $\left(C_{2}^{2},C_{2}^{-2}\right)$, respectively.

#### 2.4.2. Seidel Coefficients (Theoretical and Experimental)

The simplest metric to quantify and compare the optical quality of any optical system is the comparison of aberration coefficients. These coefficients give information about the magnitude of each aberration term. Normally, to define each aberration term separately, Zernike coefficients or Seidel coefficients are used. It should be noted that the advantage of Zernike polynomials to express *W* is that they are linearly independent; therefore, the contributions of each aberration term to the wavefront can be isolated and quantified to a large extent. In addition, when the *W* expressed in Zernike polynomials is brought to the fourth order, the following relationship between the Zernike and Seidel coefficients can be expressed

(7) $$S=6\sqrt{5}\;C_{4}^{0}\;C=3\sqrt{8}\;\sqrt{{\left(C_{3}^{1}\right)}^{2}+{\left(C_{3}^{-1}\right)}^{2}}\;A=2\sqrt{6}\;\sqrt{{\left(C_{2}^{2}\right)}^{2}+{\left(C_{2}^{-2}\right)}^{2}}$$

where *S*, *C*, and *A* indicate the spherical aberration, coma, and astigmatism coefficients, respectively.

In this work, the theoretical Seidel coefficients calculated from the Latta expressions [20,21,22,23,24] were compared with the experiments obtained from the previous conversion equations and from the Zernike coefficients obtained with the Hartmann Shack wavefront sensor. Both coefficients have also been compared with Marechal tolerances [62].

#### 2.4.3. Marechal Tolerances 

Marechal tolerances can be used to more formally assess aberration coefficients. These tolerances are based on the criteria of Lord Rayleigh, which states that the behavior of an optical system is limited only by diffraction when the phase of the wave at the output differs a maximum of λ/4 from the reference spherical wave (i.e., the ideal wavefront). From this criterion, as described by Marechal in 1970, the tolerances for each aberration term can be obtained. If we define λ, the wavelength of light, Marechal’s simplified tolerances are defined as

(8) $$S\leq 8\lambda \;C\leq \frac{4}{3}\lambda \;A\leq \frac{\lambda }{3}$$

#### 2.4.4. Root Mean Square 

Root mean square (RMS) is the most representative metric of the plane of the exit pupil. The RMS metric of optical quality is based upon the principle of measuring the optical surfaces at many points and then arriving at a single number that is a statistical measure of the departure from the ideal form [52]. In general terms, and for any type of aberration, the RMS can be defined as

(9) $$W_{RMS}=\sqrt{\frac{1}{A_{PS}}\iint^{\;}{\left[W\left(x_{p},y_{p}\right)-W_{ideal}\right]}^{2}dx_{p}dy_{p}}$$

where $A_{PS}$ is the area of the exit pupil and the $W_{ideal}$ is given as

(10) $$W_{ideal}=\frac{1}{A_{PS}}\iint^{\;}W\left(x_{p},y_{p}\right)dx_{p}dy_{p}$$

#### 2.4.5. Critical Pupil Fraction

Another metric to determine the quality of the wavefront is the pupil fraction (PF). It is defined as the fraction of the pupil area for which the optical quality is reasonably good, but not necessarily limited by diffraction. One of the methods to determine the area considered as a good pupil is to take a circular subaperture, concentric with the total pupil, within which some quality criterion is reached. This subaperture is called the critical pupil and has a critical radius ($R_{C}$) smaller than the radius of the total pupil ($R_{T}$). In this work, it has been considered as a quality criterion that the HLs must have RMS≤0.1 and is defined as

(11) $$PF={\left(\frac{R_{C}}{R_{T}}\right)}^{2}$$

#### 2.4.6. Peak to Valley

The peak-to-valley (*PV*) wavefront error is the maximum distance from the real to the ideal wavefront, in both the positive and negative directions (i.e., the distance between the maximum value and the minimum value of W). Although this is a simple method, many scientists believe that it can be misleading, since it only takes into account two points and ignores the area over which errors occur. Interestingly, the *PV* criterion became historically popular among the amateur astronomer community, who used to express the optical quality of mirrors and lenses in terms of *PV*. The *PV* wavefront error is defined as

(12) PV=max(W)−min(W)

### 2.5. Image Quality Based on Impulse Response

#### 2.5.1. Strehl Ratio

The Strehl Ratio (*SR*) is an image plane quality metric based on the impulse response (IR). It is defined as the ratio of the intensity value in the center of the image, with and without aberrations, for an optimal exit pupil size. The Strehl ratio is usually expressed as a range of numbers from 1 to 0, in which a perfect system is 1, a completely imperfect system is 0, and acceptable standards occur somewhere in between. Usually for optical instruments, it is considered as *SR* acceptable when *SR* > 0.7 or *SR* > 70%. The Strehl ratio is the relation of the central irradiations with and without variations of phase and amplitude, where the variations of phase and amplitude induce an aberrated wavefront. Mathematically, it can be defined as

(13) $$SR=\frac{I{\left(0,0\right)}_{AB}}{I{\left(0,0\right)}_{DL}}$$

where $I{\left(0,0\right)}_{AB}$ is the aberrated central irradiance and $I{\left(0,0\right)}_{DL}$ is the central irradiance limited by diffraction.

#### 2.5.2. Entropy

Entropy is a metric based on an approximation of information theory to optics. Entropy is calculated mathematically as

(14) $$H=-\underset{x,y}{\sum }ASF_{N}\left(x,y\right)\cdot lnASF_{N}\left(x,y\right)$$

where ASF is the amplitude transfer function. Entropy is the parameter that indicates how energy is distributed in the image. For an impulse response free of aberrations, the entropy will be minimal, and the concentration of light in the center is the maximum. This indicates that aberrations increase entropy because light tends to spread throughout the image. An image with a constant intensity level has the maximum entropy; therefore, the value of entropy depends largely on how the intensity or amplitude (depending on the light source used) is distributed in the image.

### 2.6. Image Quality Based on the Fourier Domain

#### 2.6.1. Cutoff Frequency

The cutoff frequency ($F_{cut}$) is a parameter that indicates the quality of the image in the Fourier domain (FD) (i.e., performs the corresponding calculations in the frequency space) and therefore evaluates the image quality. The cutoff frequency can be thought of as the spatial frequency of a grid test whose image contrast reaches zero and the details have completely disappeared. The maximum achievable cutoff frequency value corresponds to an optical system limited only by diffraction and mathematically, for an optical system that works with coherent light, is defined as

(15) $$F_{cut}=\frac{D}{2\lambda }\;cycles/rad$$

or can also be expressed in cycles/degrees

(16) $$F_{cut}=\frac{D}{2\lambda }\frac{\pi }{180}\;cycles/deg$$

The Nyquist frequency of the Hartmann–Shack sensor, calculated with Equation (1), must be taken into account.

#### 2.6.2. Modulation Transfer Function (MTF) and Area under the MTF

MTF (Modulation Transfer Function) is a measurement of the ability of an optical system to transfer modulation or contrast at a particular spatial frequency from the object to the image. It is mathematically defined as

(17) MTF=|FT(ASF)|

where ASF is the amplitude transfer function, which is the impulse response for optical systems that work with coherent light.

While the MTF captures a lot of information about an imaging system, it is desirable to describe performance with a single figure of merit, or scalar metric instead of a function. The area under the MTF has been one of the heavily researched metrics of image quality. The image quality is directly related to the integrated MTF curve between zero and the absolute limit frequency, $F_{cut\;MTF}$, (in this case, it is the Nyquist frequency because the curves are asymptotic and never cut the X-axis), which means that the richness of the information contained in the image is a function of the area under the MTF curve.

## 3. Results and Discussion

Although four types of HLs have been manufactured, this work only showed the results of two types reconstructed with two wavelengths (473 and 633 nm). The results are shown for the HLs that have the most differences between them.

### 3.1. Wavefront Aberration

**Figure 5:**
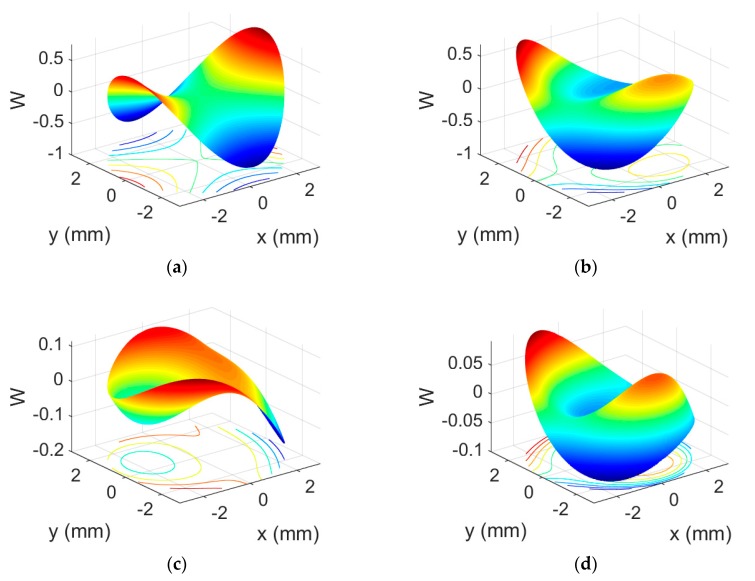
Wavefront aberration (W) of positive symmetrical (**a**) and (**b**), and negative asymmetrical (**c**) and (**d**); HLs reconstructed at 633 nm (**a**) and (**c**), and at 473 nm (**b**) and (**d**).

The three-dimensional wave aberration function, W, is represented in Figure 5. To perform this representation, the Zernike coefficients obtained with the Hartmann–Shack wavefront sensor were used.

It can be seen that negative asymmetric HLs (Figure 5c,d) had fewer aberrations than positive symmetric HLs (Figure 5a,b), and in both cases, those reconstructed at 473 nm (Figure 5b,d) had fewer aberrations than those reconstructed at 633 nm (Figure 5a,c). Furthermore, if the figures are compared with the Zernike polynomial pyramid, it can be seen that in the asymmetrical negative HLs, coma predominates, while in the symmetrical positive HLs, the astigmatic aberration predominates. This is probably due to the geometric characteristics of the recording setup. The asymmetric one was recorded with the divergent or convergent object beam in axis and the plane reference beam out of axis. The symmetric one was recorded with both the plain reference beam and the divergent or convergent object beam out of axis. The wavelength used in the recording setup was 488 nm. On the other hand, if these data are compared with those obtained theoretically from third-order expressions (i.e., Seidel’s aberrations), it can be seen that the above is true, although in the case of asymmetric HLs, the predominant theoretical aberration is spherical aberration instead of coma. These differences may be due to the possible shrinking of the material.

### 3.2. Seidel Coefficients and Marechal Tolerances

**Figure 6:**
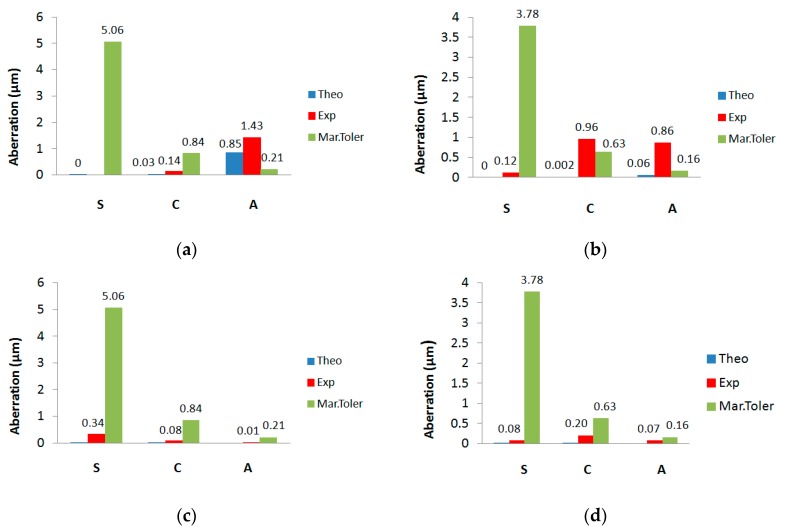
Theoretical and experimental Seidel coefficient spherical aberration (S), coma (C), and astigmatism (A) (blue and red), and Marechal tolerances (green) of the positive symmetrical (**a**) and (**b**), and the negative asymmetrical (**c**) and (**d**); HLs recorded at 633 nm (**a**) and (**c**), and at 473 nm (**b**) and (**d**).

Figure 6 shows the experimental Seidel coefficients (S, C, and A indicate spherical aberration, coma, and astigmatism coefficients) obtained from the Zernike coefficients extracted from the Hartmann–Shack wavefront sensor, the theoretical Seidel coefficients obtained from the third order aberration theory, and the aberration tolerances proposed by Marechal.

The tolerance is the upper limit value allowed for the aberration. If the aberration is greater than the tolerance, the image quality is highly affected. The tolerance must always be greater than the theoretical and experimental coefficients in order to have good image quality. 

It should be noted that off-axis aberrations such as astigmatism and coma, degrade the image quality more than the on-axis aberrations such as spherical aberration. This indicates that on-axis aberrations are more tolerable than off-axis aberrations for any optical system, in other words, the tolerance limit is higher for on-axis aberrations. In the case of asymmetric LHs, considering Figure 6c,d, we can see that all aberration coefficients were lower than the tolerances. In the case of symmetrical HLs from Figure 6a,b, Marechal’s tolerances for astigmatism were not respected. This was due to the recording geometry of this type of HLs, since the object beam impinged obliquely to the Biophotopol plate. As for the effects of the material on the image quality, it could affect the swelling or shrinkage of the material on which the hologram has been recorded. The more stable the recording material, the fewer aberrations would appear in the HL.

If the data obtained for each lens are compared by taking into account the reconstructed wavelength, it can be seen that the experimental results agreed with the theoretical ones within the precision of the approaches used. Zernike and Seidel coefficients were, in some cases, higher than the tolerances, which indicate the important presence of aberrations for both the 473 and 633 nm wavelengths. Specifically, in the case of symmetric positive HLs (Figure 6a,b), astigmatism had a higher value when illuminated with 633 nm (Figure 6a), and in both cases, it was greater than the tolerance. For the symmetric positive (+) HLs reconstructed with 473 nm (Figure 6b), we can see that the experimental coma was also greater than the tolerance. In the case of asymmetric negative (−) HLs (Figure 6c,d), we see that both theoretical and experimental coefficients were lower than the tolerances, therefore in this case, it can be said that these HLs work below the diffraction limit.

### 3.3. Optical Quality Metrics Based on Aberrations

The data obtained from the aberration-based optical quality metrics can be seen in Table 3. In this paper, an acceptable aberration value was considered to be when RMS < 0.1 [52], therefore, higher values are indicative of aberrations. It was observed that in the case of symmetric positive HLs reconstructed with 633 and 473 nm, only the experimental RMS indicated the presence of aberrations, therefore the RMS was greater than 0.1. In the case of asymmetric HLs, both the theoretical and experimental RMS had a value lower than 0.1.

PV and PF metrics are not absolute metrics, therefore, they help us to compare between the different elements. In the PV metric, a smaller value indicates smaller aberration. The values obtained for this metric indicate that its value was greater for the symmetric HLs than for the asymmetric, and the value was also greater when reconstructed with 633 nm rather than with 473 nm. On the other hand, in the PF metric, the HL must have a RMS < 0.1 in order to calculate it. For symmetrical HLs with a RMS > 0.1, we achieved a RMS < 0.1 (reasonably good optical quality, but not necessarily lower than the diffraction limit), so the critical radius must be reduced to 1.5 mm. Therefore, in general, a higher PF value indicates that a larger optical element can be manufactured, leaving the optical quality of the element intact.

### 3.4. Image Quality Based on Impulse Response and Fourier Domain

The image quality was evaluated by metrics based on the impulse response of the optical system and by metrics based on the Fourier domain. These metrics can be seen in Table 4 and Table 5. SR and normalized entropy are metrics based on the impulse response H(x, y) in amplitude because it works with coherent light. In the case of SR, a good image quality was considered when SR≥70% [52], which does not occur in the case of symmetric HL. With respect to entropy, a lower value is indicative of higher image quality. 

**Figure 7:**
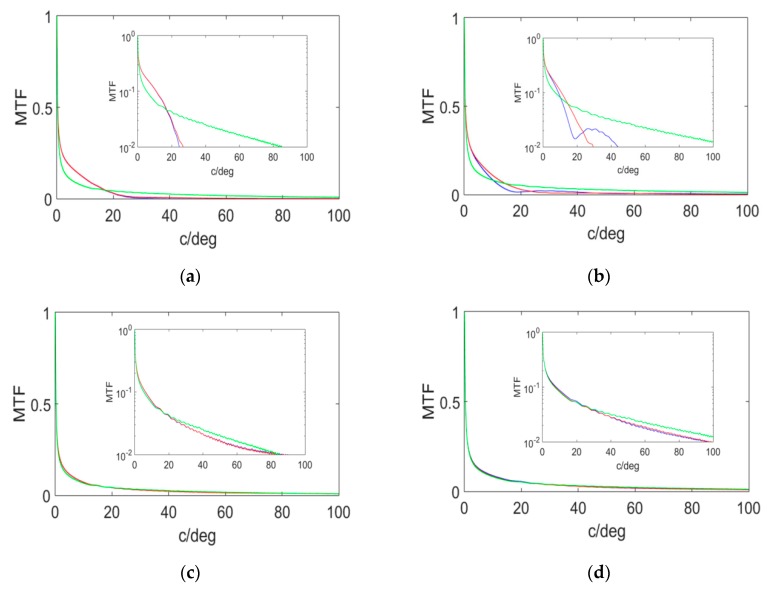
Simulated modulation transfer function (MTFs) for positive symmetrical (**a**) and (**b**), and negative asymmetrical (**c**) and (**d**); HLs reconstructed at 633 nm (**a**) and (**c**), and at 473 nm (**b**) and (**d**). A cut in x (red), cut in y (blue), and limited by diffraction (green). A zoom of these curves at a limiting resolution of 0.01 is included.

On the other hand, the cutoff frequency and the area under the MTF curve are metrics based on the frequency space. The theoretical cutoff frequency (diffraction limit) is given by Equation (16), so for the same diameter of the optical element, it will be greater for shorter wavelengths. For the experimental cutoff frequency, a cutoff on the X-axis (red), on the Y-axis (blue) of the MTF, and the MTF free of aberrations are shown in Figure 7. The curves are asymptotic and never cut the X-axis, hence, the criteria to obtain the experimental cutoff frequency is to reach an MTF value of 0.01.

It can be seen that the positive symmetric holographic lenses (Figure 7a,b) had a lower cutoff frequency than negative asymmetric holographic lenses (Figure 7c,d). It can also be seen that the HLs reconstructed at 473 nm (Figure 7b,d) had a higher cutoff frequency than the same HLs reconstructed at 633 nm (Figure 7a,c). The cutoff values can be seen in Table 4.

It was observed that the experimental cutoff frequency was lower than the theoretical one. Even so, this cut-off frequency calculated from the Zernike coefficients was obtained from the HS wavefront sensor, and we could see that there was a higher resolution than that achieved for the same HLs with a CCD sensor [57]. 

From Table 5, the highest value of the area under the MTF curve was for the asymmetric HLs reconstructed at 473 nm. In this case, the Nyquist frequency of the HS wavefront sensor must be taken into account, since working with a sensor imposes a physical limit in terms of frequency resolution.

## 4. Conclusions

For the first time, asymmetrical and symmetrical HLs were analyzed in terms of optical quality based on aberrations, image quality based on the impulse response, and image quality based on the Fourier domain by using a HS wavefront sensor as an aberrometer. Holographic lenses were stored in Biophotopol, a low toxicity photopolymer. As we have shown, there was good agreement between metrics related to optical quality, impulse response, and metrics in Fourier domain. 

This work allowed us to completely optically characterize the HL aberrations for different recording geometries (symmetric and asymmetric) and different reconstruction wavelengths (473 and 633 nm). The HL recording geometry that showed less aberration was the negative asymmetric. The other recording geometries: positive and negative symmetric, and positive asymmetric HLs showed higher aberration values. The HL with a reconstructed wavelength of 473 nm had less aberration because 473 nm is close to the recording wavelength of 488 nm. As they move away from the recording wavelength, the aberrations increase.

However, the final decision of whether to use one type of recording geometry or another will depend on the optical device in which the HL will be incorporated and its application. 

We can conclude that the negative asymmetric HL reconstructed at 473 nm had less aberration, and the calculated quality metrics allowed us to quantify the presence of aberrations in an optical system. Due to the agreement between the metrics, we can choose one indistinctly or select it depending on the information that we will need.

## Figures and Tables

**Table 1:** Parameters for the recording of the HLs.

Recording Geometry at 488 nm	Positive HL	Negative HL
Symmetrical	$\theta_{o}=17.1{}^{\circ}\;$ $\theta_{r}=-17.1{}^{\circ}$ $f\prime_{HL}=90$ mm	$\theta_{o}=17.1{}^{\circ}\;$ $\theta_{r}=-17.1{}^{\circ}$ $f\prime_{HL}=-90$ mm
Asymmetrical	$\;\theta_{o}=0{}^{\circ}\;$ $\theta_{r}=-34.2{}^{\circ}$ $f\prime_{HL}=90$ mm	$\;\theta_{o}=0{}^{\circ}\;$ $\theta_{r}=-34.2{}^{\circ}$ $f\prime_{HL}=-90$ mm

**Table 2:** Parameters for the evaluation of the HL.

Reconstruction Geometry	473 nm	633 nm
Symmetrical	$\theta_{i}=16.6{}^{\circ}\;$ $\theta_{c}=16.6{}^{\circ}$ $f\prime_{HL}=93$ mm	$\theta_{i}=22.4{}^{\circ}\;$ $\theta_{c}=22.4{}^{\circ}$ $f\prime_{HL}=70$ mm
Asymmetrical	$\theta_{i}=0{}^{\circ}\;$ $\theta_{c}=33.0{}^{\circ}$ $f\prime_{HL}=93$ mm	$\theta_{i}=0{}^{\circ}\;$ $\theta_{c}=46.8{}^{\circ}$ $f\prime_{HL}=70$ mm

**Table 3:** Optical quality metrics based on aberrations.

HL	RMSTheo (10^−4^) (μm)	RMSExp (μm)	PF	P–V (μm)
Sym (+) 633 nm	1.61	0.29	0.0625 ^1^	1.4788
Sym (+) 473 nm	0.114	0.21	0.0625 ^1^	1.3269
Asym (−) 633 nm	0.002	0.05	0.25	0.2902
Asym (−) 473 nm	0.0002	0.03	0.25	0.1776

^1^ This PF was calculated for a critical radius of 1.5 mm, which provides reasonably good optical quality, but below the diffraction limit.

**Table 4:** Image quality based on impulse response and Fourier domain.

HL	SR (%)	Normalized Entropy	Fcut MTF Dif. Lim (Green) (cycle/deg)	Fcut MTF Cut in x (Red) (cycle/deg)	Fcut MTF Cut in y (Blue) (cycle/deg)
Sym (+) 633 nm	4	18	82.7	27	24
Sym (+) 473 nm	12	7.7	110.7	29	29
Asym (−) 633 nm	80	1.8	82.7	79	79
Asym (−) 473 nm	86	1.7	110.7	99	99

**Table 5:** Image quality based on impulse response and Fourier domain.

HL	Area under MTF Cut in x (Red) (arb. units)	Area under MTF Cut in y (Blue) (arb. units)	Area under MTF Dif. Lim (Green) (arb. units)
Sym (+) 633 nm	6.2299	6.0090	7.4053
Sym (+) 473 nm	7.0814	7.0878	9.9103
Asym (−) 633 nm	7.2467	7.2208	7.4053
Asym (−) 473 nm	9.2002	9.4010	9.9103
